# Delayed diagnosis of dilated thyrotoxic cardiomyopathy with coexistent multifocal atrial tachycardia: a case report

**DOI:** 10.1186/s12872-021-01935-5

**Published:** 2021-03-04

**Authors:** Hiroyuki Yamamoto, Satoshi Monno, Keiko Ohta-Ogo, Hatsue Ishibashi-Ueda, Toru Hashimoto

**Affiliations:** 1 Department of Cardiovascular Medicine, Narita-Tomisato Tokushukai Hospital, 1-1-1 Hiyoshidai, Tomisato, Chiba 286-0201 Japan; 2 Division of Endocrinology, Department of Medicine, Hanyu General Hospital, Saitama, Japan; 3 Department of Pathology, National Cerebral and Cardiovascular Center, Suita, Japan

**Keywords:** Thyroid storm, Heart failure, Dilated thyrotoxic cardiomyopathy, Multifocal atrial tachycardia, Amiodarone

## Abstract

**Background:**

Thyroid storm (TS) is a rare but potentially life-threatening sequelae of untreated or undertreated hyperthyroidism. While TS frequently causes high-output heart failure, low-output heart failure related to dilated cardiomyopathy (DCM) is extremely rare. Tachycardia is a common clinical presentation of TS, and β1-selective blockers are the first-line agents for treating TS-associated tachycardia. However, given that β-blockers have negative chronotropic and negative inotropic effects, amiodarone may be safe and effective for the treatment of TS-induced tachyarrhythmia in patients with moderate to severe heart failure. While long-term amiodarone administration causes hypothyroidism, or less frequently, hyperthyroidism, little is known about the effects of short-term amiodarone administration on thyroid function.

**Case presentation:**

A 31-year-old healthy woman presented with worsening dyspnoea. She was tachycardic with multifocal atrial tachycardia (MAT) of 184 beats/min, confirmed by electrocardiogram. Echocardiographic findings were consistent with DCM, with an ejection fraction of 20%. Thus, she was initially diagnosed with acute heart failure due to DCM with coexistent MAT. Tachycardia persisted despite cardioversion attempts and treatment with multiple anti-arrhythmic drugs. Consequently, she rapidly progressed to cardiogenic shock and respiratory decompensation, which required intubation and an intra-aortic balloon pump support. Moreover, the undiagnosed Graves' disease, lack of suspicion, and postponed analysis of thyroid function tests led to a delayed diagnosis of TS. Amiodarone, which was initiated for MAT, unexpectedly ameliorated thyrotoxicosis, resulting in a euthyroid state and the patient’s significantly improved condition and cardiac function. She was discharged on day 40. Finally, she underwent total thyroidectomy; thyroid pathology was consisting with Graves' disease. Her postoperative course was uneventful.

**Conclusions:**

Herein, we describe a case of delayed diagnosis of dilated thyrotoxic cardiomyopathy with coexistent MAT. The patient required intensive care due to the catastrophic sequelae and was successfully treated with amiodarone. This is the first case report of TS-associated MAT and highlights the clinical importance of high suspicion of TS in de novo heart failure with any tachyarrhythmia or DCM of unknown etiology and the potential effects of short-term amiodarone administration in the treatment of TS.

## Background

Thyroid storm (TS), a sudden and life-threatening exacerbation of thyrotoxicosis, can lead to irreversible multiple organ failure if left untreated. Often, TS causes high-output heart failure (HF) with coexistent atrial fibrillation or sinus tachycardia due to hyperdynamic status and decreased systemic vascular resistance. However, low-output HF related to dilated cardiomyopathy (DCM) is rare [1].

Tachycardia is a common clinical presentation of TS. As mortality and severity in patients with TS significantly increases as resting heart rate increases [2], and thyroid hormones increases the number of beta-adrenergic receptors [3], national and international guidelines recommend β1-selective blockers (landiolol, esmolol, or bisoprolol) as first-line agents for the treatment of TS-associated tachycardia [4]. However, treating TS-associated tachycardia remains challenging in patients with moderate to severe HF, given that β-blockers have negative chronotropic and negative inotropic effects.

Amiodarone, a class III potent anti-arrhythmic drug, is effective for controlling atrial tachyarrhythmia, regardless of the hemodynamic status [5], and thus is recommended for treating tachyarrhythmia in TS during a left ventricular systolic dysfunction (LVSD). Moreover, amiodarone can directly impact thyroid function due to its high iodine content [6]. While long-term amiodarone administration is reported to cause hypothyroidism, or less frequently hyperthyroidism [7], little is known about the effects of short-term amiodarone administration on thyroid function.

## Case presentation (Table 1)

**Fig. 1:**
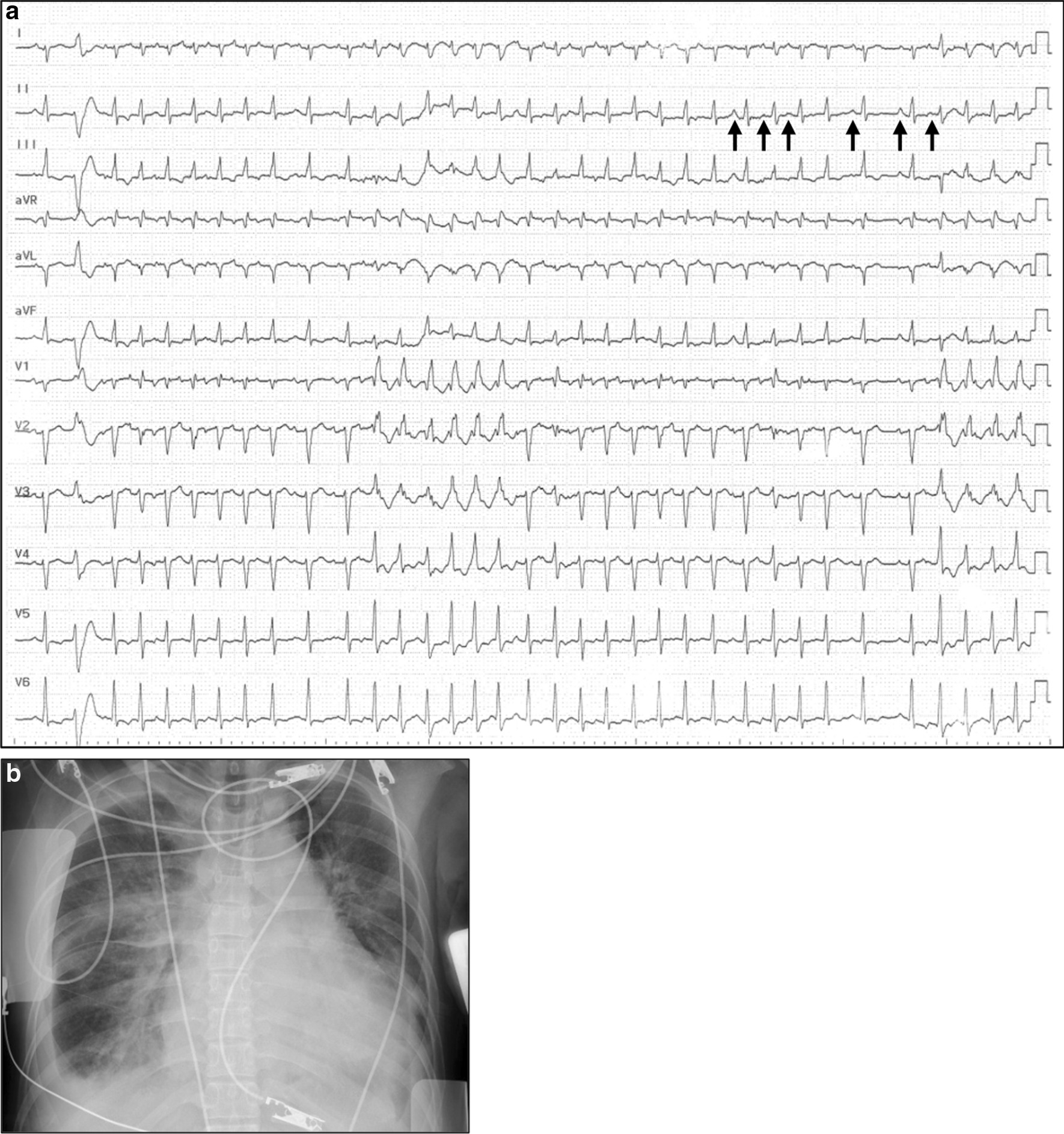
Representative images depicting the findings of the electrocardiogram (**a**) and chest radiography (**b**). Electrocardiogram showing isoelectric baseline between P waves, and rapid, irregular rhythm with at least three distinctive P-wave morphologies (arrows; best seen in the II leads). Notably, some P waves were aberrantly conducted to the ventricles, and premature ventricular contraction was also detected. The heart rate was 184 beats/min (**a**)

**Fig. 2:**
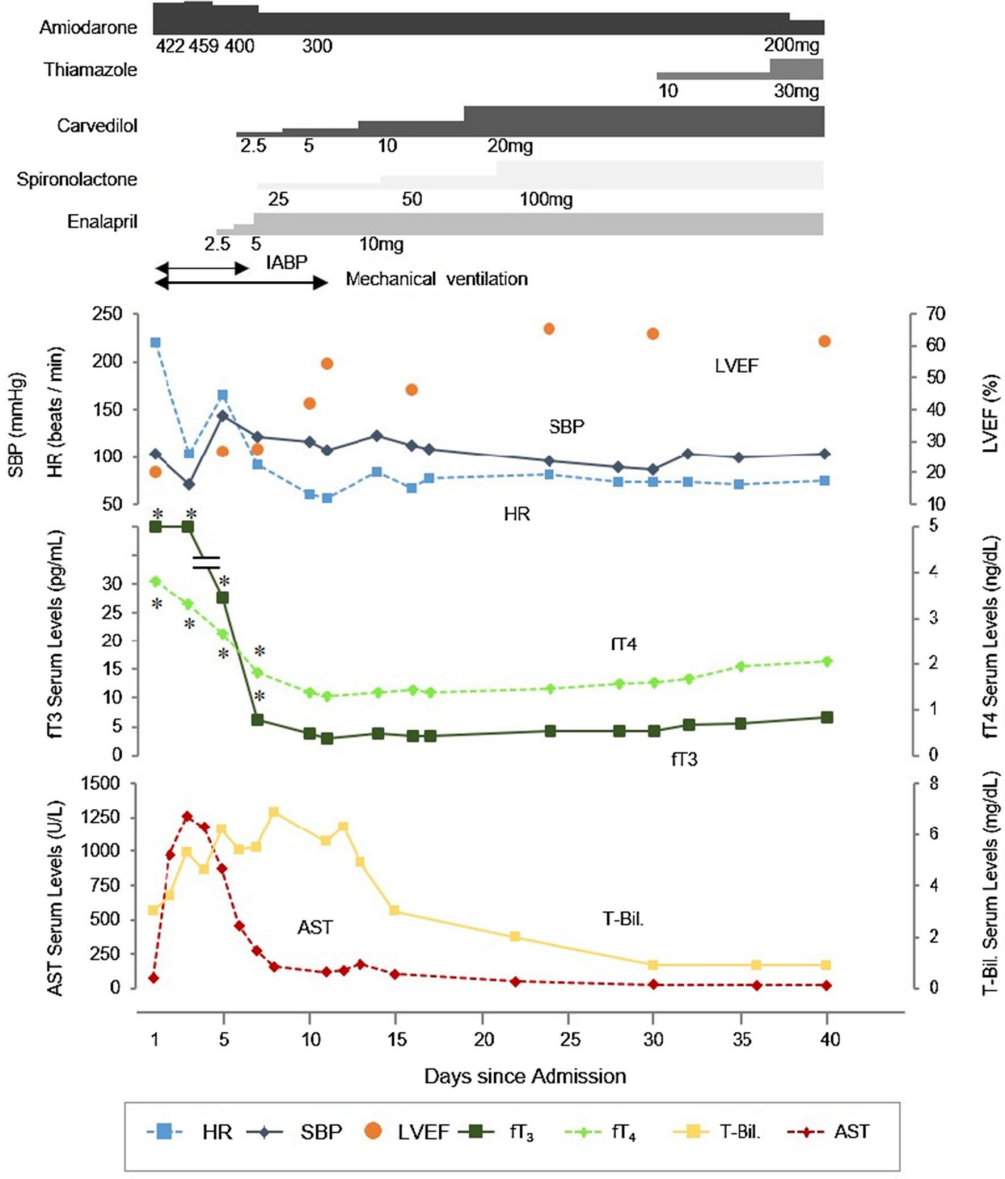
Clinical course after admission. HR: Heart rate, SBP: Systolic blood pressure, LVEF: Left ventricular ejection fraction, fT3: Free T3, fT4: Free T4, T-Bil: Total bilirubin, AST: Aspartate-aminotransferase. *Data were retrospectively measured using stored blood samples obtained on admission day 1 through day 7

A 31-year-old woman was admitted to the emergency department with worsening dyspnoea. She begun to feel dyspnoea on effort 10 days prior and 4 days before admission, the symptom worsened accompanied by a non-productive cough on deep breathing. However, she had no subjective symptoms of palpitations, no underlying cardiac disease, and was not receiving any regulation medication or consuming supplements. Upon initial examination, the patient was alert, oriented, and afebrile, with a blood pressure of 137/78 mmHg, tachycardia of 244 beats/min, and tachypnoea of 36 breaths/min. Cardiovascular auscultation revealed cardiac gallop rhythm and bilateral crackles. Electrocardiogram revealed three or more distinctive P**-**wave morphologies, plus tachycardia with a heart rate of 184 beats/min (Fig. 1a). The P**-**wave morphologies were different from the normal sinus P**-**wave morphology in the previous electrocardiogram performed 5 years earlier (Additional file 1), suggesting multifocal atrial tachycardia (MAT). Chest radiography revealed cardiomegaly with pulmonary congestion (Fig. 1b), and echocardiography revealed left ventricular dilatation and generalized severe hypokinesis with an ejection fraction of 20%, suggesting DCM. Further, laboratory tests revealed elevated levels of aspartate aminotransferase (84 U/L, reference: 13–30 U/L), alanine aminotransferase (80 U/L, reference: 7–23 U/L), total bilirubin (3.0 mg/dL, reference: 0.4–1.5 mg/dL), cardiac troponin I (35.6 pg/mL, reference: < 26.2 pg/mL), and brain natriuretic peptide (1,524.4 pg/mL, reference: < 18.4 pg/mL). Tachycardia persisted despite intravenous administration of adenosine triphosphate, verapamil, and landiolol, and multiple cardioversion attempts were ineffective. The patient rapidly progressed to cardiogenic shock and respiratory decompensation, necessitating transfer to the intensive care unit, intubation, and inotropic support. She received infusions of noradrenaline (0.04 µg/kg/min), dobutamine (2 µg/kg/min), and milrinone (0.125 µg/kg/min). Mildly elevated circulating cardiac enzymes were noted and acute coronary syndrome was suspected. However, urgent coronary angiography was unremarkable and an intra-aortic balloon pump (IABP) was inserted. Amiodarone was started for refractory tachyarrhythmia. The intravenous dose was started with 125 mg administered over 10 min, followed by an infusion of 0.8 mg/min for 6 h, and then a maintenance infusion of 0.4 mg/min for the next 18 h. A subsequent oral dose was started at 400 mg/day and gradually reduced to 200 mg/day. Incessant tachyarrhythmia finally reverted to sinus rhythm and resulted in a significant heart rate reduction. Administration of a vasopressin V2-receptor antagonist (tolvaptan 15 mg/day) with loop diuretics (azosemide 30 mg/day) was initiated to ameliorate congestion. In addition, anticoagulation with intravenous heparin (19,200 units/day) was initiated to prevent intracardiac thrombus formation, and low-dose pimobendane (1.25 mg twice daily) was used to enhance left ventricular systolic function. She remained hemodynamically stable with support from the IABP and was progressively weaned from the vasoactive agents. After confirming the patient’s condition to be euvolemia, anti-failure therapy was started at extremely low doses (enalapril, spironolactone, and carvedilol), which were carefully and gradually increased based on the patient’s tolerance (Fig. 2). IABP was weaned on day 7. After confirming that there was no re-exacerbation of HF following the introduction of anti-failure medication, the patient was weaned from the ventilator and transferred to the general ward on day 11.

**Fig. 3:**
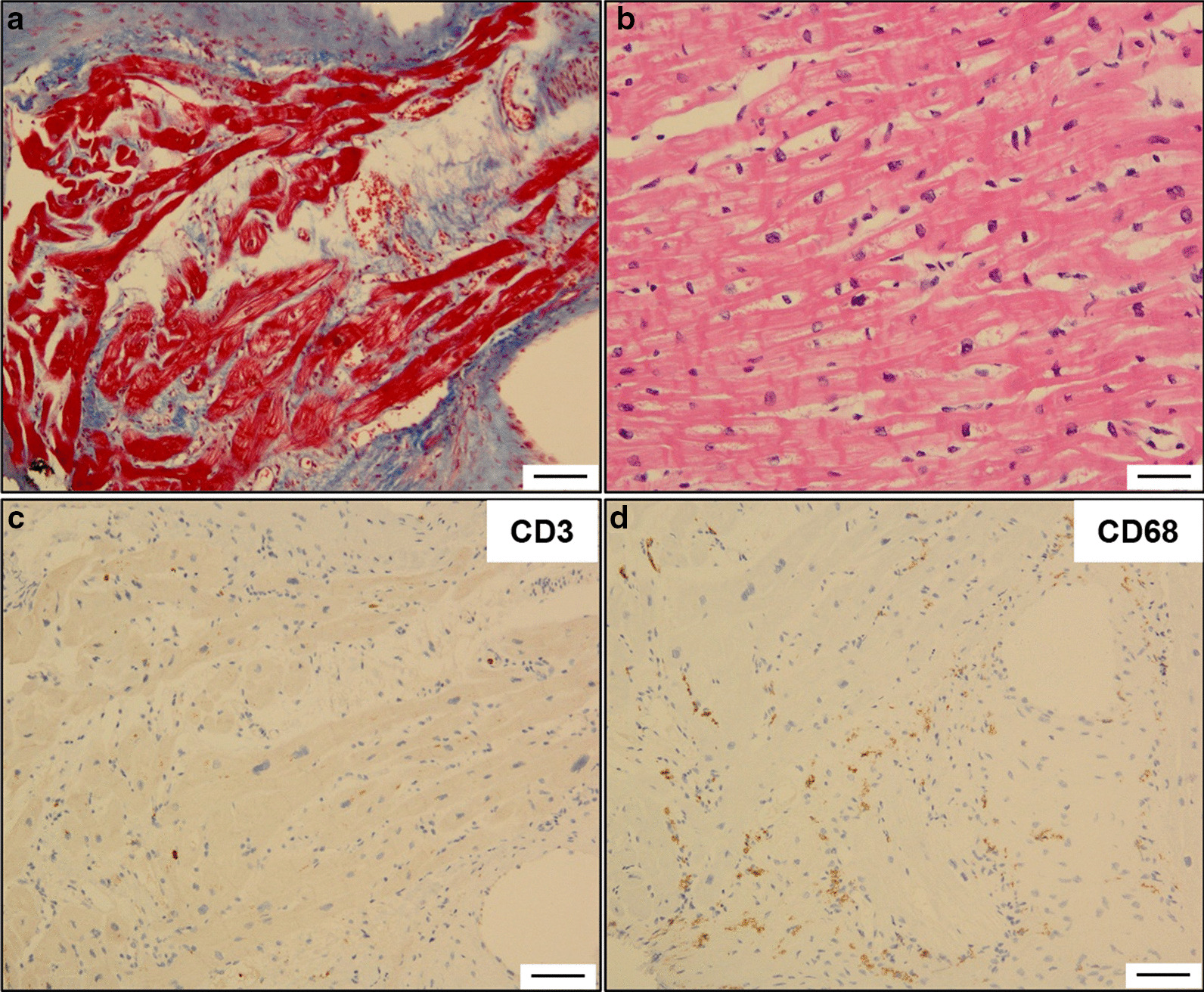
Endomyocardial biopsy of the left ventricle. Photomicrograph showing moderate to severe endocardial thickening, interstitial and perivascular fibrosis, and focal interstitial edema (scale bar: 100 µm) (**a**). High-power view with hematoxylin–eosin stain showing moderate hypertrophy of myocytes with no remarkable myocyte loss (scale bar: 50 µm) (**b**). Some CD3 + T**-**lymphocytes can be observed (scale bar: 100 µm) (**c**). CD68 + macrophages can be observed sporadically in the interstitial fibrosis area (scale bar: 100 µm) (**d**)

Diagnostic endomyocardial biopsy (EMB) was performed on day 8. Photomicrograph revealed moderate myocyte hypertrophy with moderate interstitial fibrosis and a few interstitial inflammatory infiltrates without associated myocyte necrosis (Fig. 3). The counts of CD3 + T-lymphocytes and CD68 + macrophages were 2.2 and 23.9 cells/mm^2^, respectively; this corresponded to 6.2 leucocytes/mm^2^, including up to 4 monocytes/mm^2^, which did not fulfill the proposed immunohistochemical criteria for myocarditis (≥ 14 leucocytes/mm^2^, including up to 4 monocytes/mm^2^ and CD3 + T-lymphocytes ≥ 7 cells/mm^2^) by World Heart Federation and European Society of Cardiology. Furthermore, late gadolinium-enhanced cardiac magnetic resonance imaging showed no abnormalities, indicating the absence of myocardial necrosis, supporting this notion.

In addition, a workup for the unexplained tachycardia was performed. We performed thyroid function tests using stored blood samples obtained on admission day 1 through day 7 (Table 2). Strikingly, thyroid studies using pre-amiodarone treatment sera on admission demonstrated undetectable thyroid-stimulating hormone (TSH) levels (reference: 0.35–4.94 mIU/L), and markedly elevated free T3 and T4 (> 30 pg/mL, reference: 1.71–3.71 pg/mL; 3.81 ng/dL, reference: 0.7–1.48 ng/dL, respectively), strongly suggesting thyrotoxicosis.

**Table 1:** Timeline

Timeline	
14 days prior to presentation	The patient experienced diarrhoea and nausea
10 days prior to presentation	She began to feel dyspnoea on effort
4 days prior to presentation	She experienced worsening dyspnoea and a nonproductive cough on deep breathing
*On emergent presentation*	
6:40:00 PM	She presented with worsening dyspnoea at the emergency department. Electrocardiogram showed MAT. Echocardiography revealed left ventricular dilatation and severe hypokinesis; the patient was diagnosed with dilated cardiomyopathy
6:44:00 PM	She was treated for MAT with intravenous administration of adenosine triphosphate, verapamil, and landiolol, as well as multiple cardioversions, which were ineffective
8:12:00 PM	She rapidly progressed to cardiogenic shock and respiratory decompensation, which required intubation and inotropic support
9:35:00 PM	Emergent coronary angiography was unremarkable. An IABP was inserted
11:59:00 PM	Amiodarone was started for refractory MAT
Day 3	IABP and administration of amiodarone successfully suppressed the recurrence of MAT
Day 5	She was weaned from the vasoactive agents, and anti-failure therapy was carefully induced
Day 7	She was weaned off IABP
Day 8	Endomyocardial biopsy was performed A workup for the unexplained tachycardia led to the correct diagnosis of thyroid storm
Day 11	Extubation and cardiac rehabilitation
Day 31	Thiamazole was induced
Day 38	Follow-up echocardiography demonstrated significant improvements in left ventricular systolic function and reverse remodelling
Day 40	Discharged to home
Regular follow-up	She received treatment at our outpatient clinic to establish clinical euthyroidism
Day 131	Total thyroidectomy was performed; thyroid pathology was consistent with Graves' disease
6-month follow-up 48-month follow-up	Full recovery of the LVSD was observed She remained clinically stable

MAT: Multifocal atrial tachycardia, IABP: Intra-aortic balloon pump, LVSD: Left ventricular systolic dysfunction

**Table 2:** Trend of thyroid function tests

Laboratory findings	Results					Reference value
Thyroid function	On admission*	Day 3*	Day 5*	Day 7*	Day 10	
TSH	< 0.01 mIU/L**	< 0.01 mIU/L	< 0.01 mIU/L	< 0.01 mIU/L	< 0.01 mIU/L	0.35–4.94 mIU/L
fT3	> 30 pg/mL**	> 30 pg/mL	27.61 pg/mL	6.25 pg/mL	3.83 pg/mL	1.71–3.71 pg/mL
fT4	3.81 ng/dL**	3.32 ng/dL	2.66 ng/dL	1.81 ng/dL	1.38 ng/dL	0.7–1.48 ng/dL
TgAb					531 IU/mL	0–28 IU/mL
TPOAb					45 IU/mL	0–16 IU/mL
TRAb					11.5 IU/L	0–2.0 IU/L
TSAb					651%	0–120%

TSH: Thyroid-stimulating hormone, fT3: Free triiodothyronine, fT4: Free thyroxine, TgAb: Anti-thyroglobulin antibody, TPOAb: Anti-thyroid peroxidase antibody, TRAb: TSH receptor antibody, TSAb: Thyroid-stimulating antibody

*Data were retrospectively measured using stored blood samples obtained on admission Day 1 through Day 7

**Data using pre-amiodarone treatment sera

**Fig. 4:**
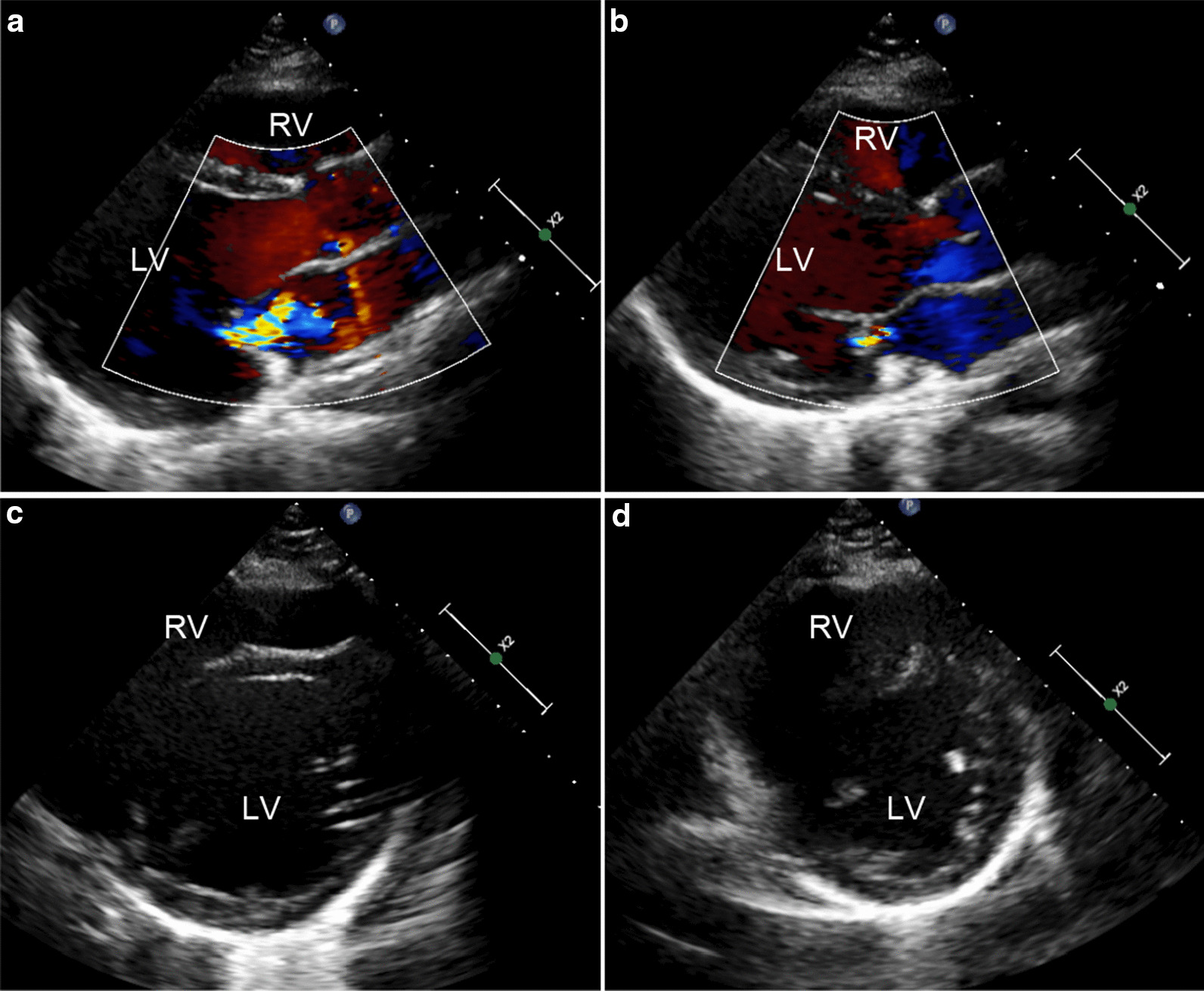
Effects of amiodarone treatment on echocardiographic parameters. Color Doppler TTE in parasternal long-axis view (**a**, **b**), and TTE in parasternal short-axis view (**c**, **d**). Echocardiography (day 4) demonstrates severe dilated cardiomyopathy with moderate MR (LVEF, 27%; LVDD, 61 mm) (**a**, **c**). Follow-up echocardiography (day 38) demonstrates significant improvement in ventricular function and MR (LVEF, 61%; LVDD, 37 mm) (**b**, **d**). LV: Left ventricle, LVEF: Left ventricular ejection fraction, LVDD: Left ventricular diastolic diameter, MR: Mitral regurgitation, RV: Right ventricle, TTE: Transthoracic echocardiography

In consultation with an endocrinologist, a thorough physical examination revealed mild exophthalmos and thyromegaly, which could not be recognised during the initial physical examination. Graves' disease (GD) was suspected as a cause of the thyrotoxicosis. In addition, antibodies to thyrotropin receptor (TRAb) (11.5 IU/L, reference: 0–2.0 IU/L), and thyroid-stimulating antibodies (TSAb) (651%, reference: 0–120%) were detected, further supporting this notion (Table 2). After a careful interview, the subject revealed about a two-week history of diarrhoea and nausea before admission, suggesting preceding gastroenteritis. The patient fulfilled the Japan Thyroid Association diagnostic criteria for TS. Notably, we recognized that amiodarone treatment had unexpectedly controlled TS (Fig. 2). Her clinical status improved during her hospitalization. However, the patient’s thyroid hormone levels began to increase on day 14, suggesting the escape phenomenon. Thiamazole (10 mg/day) was started on day 31. As the patient was a staff nurse of our hospital, she was permitted and wished to continue her hospitalization and rehabilitation. Thus, she was finally discharged on day 40, when serial echocardiography showed significant improvements in the left ventricular systolic function and reverse remodeling (Fig. 4; Additional files 2, 3, 4, 5). The patient subsequently received treatment at our outpatient clinic to establish clinical euthyroidism. She remained clinically stable during a 2-month follow-up, and the results of thyroid function tests were as follows: undetectable TSH, free T3 (4.38 pg/mL), free T4 (1.35 ng/dL), TRAb (5.1 IU/L), and TSAb (245%).

Ultimately, total thyroidectomy was performed because she wished to become pregnant. The resected thyroid gland was markedly enlarged and weighed 122 g. Thyroid pathology was consistent with GD. The postoperative course was uneventful. Full recovery of the LVSD was observed at the 6-month follow-up. Even after subsequent tapering and discontinuation of beta-blockers, she remained clinically stable at the 48-month follow-up.

## Discussion

The pathophysiologic mechanisms of TS are elusive and diagnosing TS remains challenging since its diagnosis is based upon the presence of pathognomonic clinical features consistent with thyrotoxicosis [2]. We initially considered that MAT was common in acute HF and was presumably attributed to the compensation of LVSD. Moreover, neither high-grade fever nor central nervous system manifestations, both common and specific to TS, were observed in this case. Thus, undiagnosed GD, lack of suspicion, and postponed analysis of thyroid function tests led to the delayed diagnosis of TS. In addition, elevated circulating cardiac enzymes prompted coronary angiography with suspicion of myocardial ischemia. However, coronary angiography could have been avoided if thyroid hormone screening at the initial examination had confirmed the corrective diagnosis of TS. Actually, the ACCF/AHA guidelines strongly recommend including thyroid function tests for all patients with HF as their initial workup, which also applies to patients with DCM [8, 9]. Consequently, our patient did not receive the best guideline-mediated treatment for TS, including anti-thyroid drugs, β-blockers, iodine therapy, and glucocorticoids [4]. Nevertheless, she could recover from the catastrophic sequelae through intensive care with amiodarone treatment. Our case might provide several valuable lessons as follows:

First, our case presented as dilated thyrotoxic cardiomyopathy (DTC), which had a striking recovery as evidenced by the achievement of an euthyroid state. DTC is an uncommon phenotype of TS, and has a prevalence of less than 1% in thyrotoxic patients [1]. Multiple factors, such as genomic or non-genomic effects of thyroid hormone on the heart and blood vessels, may be involved in cardiomyopathy [10], and uncontrolled thyrotoxicosis, autoimmune, or inflammatory reactions can cause DTC [11]. Besides, persistent tachycardia can cause DCM as a result of impaired myocardial contraction due to diminished activity of the Na/K-ATPase pump, and downregulation of beta-adrenergic receptors [12].

Given that GD is an autoimmune thyroid disease, and that thyroid hormone receptors have been identified in human cardiomyocytes [13], it is plausible that autoimmune-mediated inflammatory responses are directly responsible for the development of DTC. Indeed, autoimmune lymphocytic myocarditis has been demonstrated to be a major causative factor of DTC [14, 15]. However, a previous biopsy study reported that most of the biopsy tissue from cases of GD with LVSD present secondary DCM, characterized by myocyte hypertrophy and interstitial fibrosis [16]. Our case revealed similar histological features, and did not comply with the histological diagnostic criteria for myocarditis [17]. Instead, tachycardia-induced cardiomyopathy was considered to be the most likely mechanism of cardiomyopathy in our case owing to the persistence of incessant tachycardia, exclusion of other possible causes of HF, and the complete recovery of LVSD following the restoration of sinus rhythm and heart rate control [12].

In our case, the EMB was not performed during the very acute phase of hospitalization. As recommended in the guideline, EMB should have been performed as soon as possible to exclude fulminant myocarditis and identify the presence of treatable myocarditis such as eosinophilic myocarditis and giant cell myocarditis as the possible cause of unexplained HF with cardiogenic shock or fatal arrhythmia [17]. Importantly, the reversibility of thyrotoxic cardiomyopathy requires an early recognition and proper treatment of hyperthyroidism [18].

Second, to the best of our knowledge, ours is the first report of a case of TS-associated MAT. The most common rhythm disturbance in TS is sinus tachycardia or atrial fibrillation [19]. The postulated mechanism of tachycardia is through excessive beta-adrenergic activity due to an increased number of beta-adrenergic receptors in hyperthyroidism [3]. MAT is a relatively uncommon arrhythmia, recognized in 0.05% to 0.3% of electrocardiograms in hospitalized patients [20]. While the majority of MAT cases are associated with significant lung disease, such as acute respiratory failure, pulmonary artery hypertension, and chronic obstructive pulmonary disease, MAT is also related to other conditions, including congestive HF, electrolyte imbalance, and drugs, such as isoproterenol and aminophylline. Although the mechanism of MAT remains poorly understood, the most plausible pathophysiological mechanisms include re-entry, abnormal automaticity, and triggered activity.

In the current case, TS, probably triggered by the preceding infection, led to severe hypoxia, acidosis, overactivation of the sympathetic nervous system, and abnormal automaticity. Moreover, secondary pulmonary hypertension caused by LVSD, and the subsequent increased atrial pressure, were also likely to have contributed to the increase in automaticity. Delayed atrial afterdepolarizations caused by intracellular calcium overload in DTC can lead to triggered activity, which is capable of initiating and maintaining cardiac arrhythmias. Thus, these factors might contribute to MAT, which is a heart rate above 180 beats/min. However, in the current case, given the lack of response to multiple electrical cardioversion trials, re-entry was unlikely to be related to the genesis of MAT.

Lastly, amiodarone administration was useful for both the management of TS-associated MAT and the amelioration of thyrotoxicosis.

Long-term amiodarone administration may cause hypothyroidism, or less frequently, hyperthyroidism [7]. The inhibitory effect of amiodarone on thyroid function has generally been reported to occur from 2 weeks to approximately 3 years after the initiation of amiodarone treatment. However, the effects of short-term amiodarone administration remain elusive. To the best of our knowledge, our case is the first report of the effects of short-term administration of amiodarone in TS. Amiodarone can effectively control the ventricular rate, and convert to and maintain sinus rhythm without hemodynamic instability against atrial tachyarrhythmia [5]; in our case, amiodarone was also found to be useful for TS-associated MAT.

Moreover, amiodarone has various effects on thyroid function because of its high iodine content and is reported to directly affect the thyroid gland [6]. Amiodarone ameliorates hyperthyroidism by blocking the conversion of T4 to T3, T4 entry into the peripheral tissues, and beta-adrenergic receptors. Thyroid hormone release can also be inhibited by amiodarone because its high iodine content leads to reduced thyroidal iodine uptake. Therefore, we believe that amiodarone may have played a major role in the treatment of TS, based upon the fact that the abnormally high thyroid hormone levels rapidly improved following amiodarone treatment, leading to the rescue of the patient from a fatal situation. However, anti-thyroid drugs were ultimately needed to control the thyrotoxicosis due to the escape phenomenon from the Wolff-Chaikoff effect [21] (Fig. 2). Furthermore, the iodinated contrast media received on admission might have had an additional effect on the improvement of TS since iodine is one of the therapeutic options for TS to inhibit excessive thyroid hormone release. Besides, it is recommended that iodine treatment should be delayed at least one hour after thionamide treatment to prevent undesired tyrosine residue iodination and enrichment of thyroid hormone stores [4]. Thus, there remain many unanswered questions, and further investigation is needed to assess whether the effects of short-term amiodarone administration are beneficial or harmful for patients with TS.

## Conclusion

Herein, we describe a case of delayed diagnosis of DTC with coexistent MAT, successfully treated with amiodarone. TS, which can present as DCM or MAT, is a rare but life-threatening cardiac disease, which can be cured if treated appropriately at an early stage. Therefore, clinicians should always consider the possibility of this rare clinical entity in patients with de novo HF, with any tachyarrhythmia or DCM of unknown etiology. Moreover, our case highlights the potential effects of short-term amiodarone administration in the treatment of TS.

## Supplementary Information

**Additional file 1.** Electrocardiogram performed during physical examinations 5 years prior. Electrocardiogram showing a normal sinus rhythm with inverted Twaves in leads II, III, and aVF.

**Additional file 2.** Color Doppler t ransthoracic echocardiography in parasternal long axis view onday 4. Parasternal long axis view with color Doppler showing severelydilated, diffusely hypokinetic left ventricle with moderate mitral regurgitation.

**Additional file 3.** Color Doppler transthoracic echocardiography in parasternal long-axis view on day 38. Parasternal long-axis view with color Doppler showing significant improved left ventricular function, and amelioration of mitral regurgitation.

**Additional file 4.**. Transthoracic echocardiography in parasternal short-axis view on day 4. Parasternal short-axis view showing severely dilated, diffusely hypokinetic left ventricle.

**Additional file 5.**. Transthoracic echocardiography in parasternal shot-axis view on day 38. Parasternal short-axis view showing significant improved left ventricular function.

## Data Availability

Data sharing is not applicable to this article as no datasets were generated or analyzed during the current study.

## Abbreviations

TS: Thyroid storm
HF: Heart failure
DCM: Dilated cardiomyopathy
LVSD: Left ventricular systolic dysfunction
MAT: Multifocal atrial tachycardia
IABP: Intra-aortic balloon pump
TSH: Thyroid-stimulating hormone
EMB: Endomyocardial biopsy
GD: Graves' disease
TRAb: Antibodies to thyrotropin receptor
TSAb: Thyroid-stimulating antibodies
DTC: Dilated thyrotoxic cardiomyopathy
