# Supplementary figures and images for: Delayed diagnosis of dilated thyrotoxic cardiomyopathy with coexistent multifocal atrial tachycardia: a case report
Source: BMC Cardiovasc Disord. 2021 Mar 4;21:124. doi: 10.1186/s12872-021-01935-5 (PMC7931980; doi:10.1186/s12872-021-01935-5)

## Slide 1
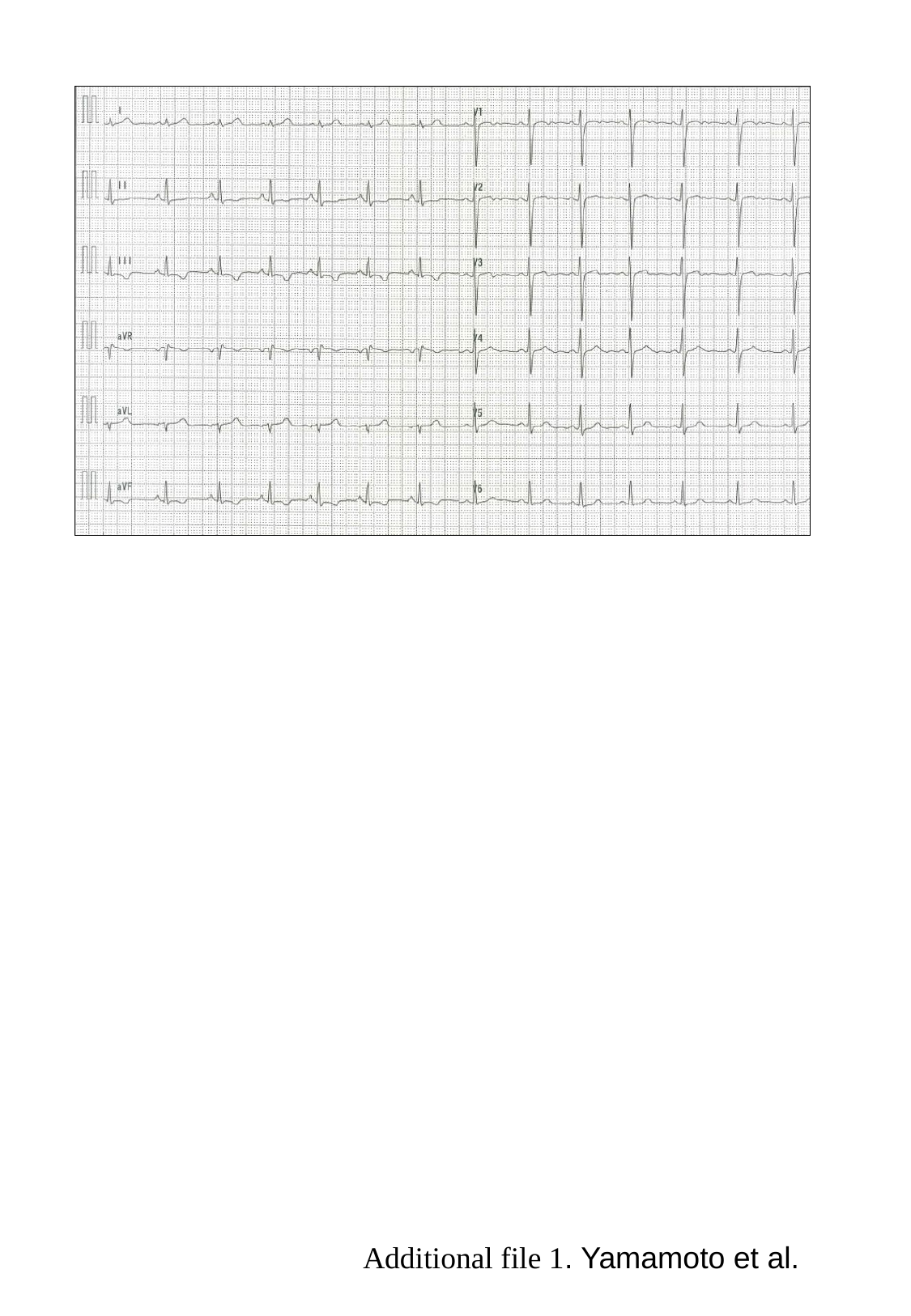

Additional file 1. Yamamoto et al.

Supplement: Supplementary file 1 — Additional file 1. Electrocardiogram performed during physical examinations 5 years prior. Electrocardiogram showing a normal sinus rhythm with inverted Twaves in leads II, III, and aVF. [file 12872_2021_1935_MOESM1_ESM.pptx]
